# Extrusion-Assisted Formation of Rice Starch–Propyl Gallate Complexes: Structural Characteristics, Antioxidant Activity, and In Vitro Digestibility

**DOI:** 10.3390/foods15111880

**Published:** 2026-05-26

**Authors:** Simeng Ma, Zhuanghong Wang, Honghao Fan, Hai He

**Affiliations:** 1 Key Laboratory of Tropical Translational Medicine of the Ministry of Education, School of Public Health, Hainan Academy of Medical Sciences, Hainan Medical University, Haikou 571199, China; 2 NJUST-YX Artificial Intelligence Biomedical Technology Innovation Center, Nanjing University of Science and Technology, Nanjing 210094, China

**Keywords:** propyl gallate, rice starch, antioxidant activity, extrusion cooking, in vitro digestibility

## Abstract

Propyl gallate (PG) is an effective food antioxidant, but its performance in food systems may be limited by poor water compatibility and processing instability. In this study, rice starch was used as a carrier matrix to prepare starch–PG complexes by extrusion cooking, and the effects of PG incorporation on starch structure, antioxidant activity, and in vitro digestibility were evaluated. Starch was blended with PG at 0, 25, 50, and 100 mg/g and processed by extrusion, and the resulting samples were characterized by complex index analysis, small-angle X-ray scattering, Fourier-transform infrared spectroscopy, solid-state carbon-13 nuclear magnetic resonance, X-ray diffraction, pasting and rheological measurements, 1-diphenyl-2-picrylhydrazyl (DPPH) radical-scavenging assay, in vitro digestibility, and density functional theory calculation. Extrusion disrupted the native semi-crystalline structure of starch, while PG incorporation promoted complex formation, with the highest complex index (88.28%) observed at 50 mg/g PG. Structural analyses indicated increased short-range order, higher single-helical content, and the development of V-type crystalline features in the PG-containing extruded starches. These starches also retained DPPH radical-scavenging activity and showed slower in vitro starch hydrolysis, with resistant starch increasing to 25.78%. Overall, extrusion cooking appears to be a feasible approach for preparing starch–PG complexes that preserve antioxidant functionality and reduce in vitro digestibility.

## 1. Introduction

Lipid oxidation is widely recognized as a primary cause of quality deterioration in food systems during processing and storage [1]. Essential nutrients such as vitamins and unsaturated fatty acids are degraded by oxidative processes, which also generate volatile compounds that cause rancidity and off-flavors. Because these reactions directly affect food quality, safety, and consumer acceptance, controlling lipid oxidation has become a critical challenge in the food industry [2]. Antioxidants are therefore widely incorporated into food formulations to inhibit free radical reactions and delay oxidative degradation [3]. Antioxidants, including synthetic phenolic antioxidants, vitamins, carotenoids, tocopherols, and plant-derived polyphenols, are widely used to delay oxidative degradation in foods. In recent years, natural antioxidants have attracted growing interest due to consumer preference for clean-label and plant-derived functional ingredients [4]. Enhancing the stability and delivery efficiency of antioxidant compounds for functional food applications has also received greater focus in recent years [5].

Among the antioxidants used in food systems, propyl gallate (PG) is a widely applied phenolic antioxidant known for its strong radical-scavenging ability. PG is commonly added to lipid-rich products such as edible oils, fried foods, and snacks to prevent oxidative deterioration and extend shelf life [6,7]. However, the practical application of PG is limited by several physicochemical constraints, including low water solubility and susceptibility to thermal or oxidative degradation during food processing [8]. These limitations can reduce its stability and antioxidant efficiency in complex food matrices. Consequently, strategies that enhance the stability and functionality of PG are required to improve its performance in food systems [6,7].

Encapsulation technology has emerged as an effective approach for protecting sensitive bioactive compounds and improving their functional performance. By incorporating active ingredients within protective matrices, encapsulation systems can shield them from environmental stresses such as oxygen, heat, and moisture during food processing and storage [9]. In addition to improving stability, encapsulation enables the controlled release of bioactive compounds, potentially enhancing their bioavailability and functional efficacy [10]. Various materials have been investigated as encapsulation carriers, including proteins, lipids, synthetic polymers, and polysaccharides. Polysaccharides, especially starch, have garnered considerable interest among these substances due to their natural origin, affordability, and suitability for food systems [9].

One of the most prevalent polysaccharides in nature, starch has been extensively studied as a vehicle for bioactive substances. Compared with protein- or lipid-based carriers, starch matrices generally exhibit superior thermal stability, making them suitable for heat-intensive food processing technologies [11]. In addition to serving as a physical encapsulation matrix, starch can interact with hydrophobic molecules to form molecular complexes. Specifically, single-helical conformations of amylose chains may provide hydrophobic cavities that can accommodate small guest molecules such as lipids and phenolic compounds. These amylose inclusion complexes provide an effective mechanism for stabilizing bioactive compounds within starch matrices [12].

From a sustainability perspective, rice starch can also be recovered from rice-processing by-products and food-waste streams, such as broken rice, rice flour residues, and starch-rich waste generated during rice-based food production. Valorizing these underutilized resources as starch sources may reduce food-processing waste while providing low-cost raw materials for chemical or physical modification [13]. Therefore, waste-derived rice starch could serve as a promising starting material for preparing starch-based delivery systems and functional ingredients.

Previous studies have shown that starch can interact with various phenolic compounds, including gallic acid, catechin, quercetin, ferulic acid, and other polyphenols, through hydrogen bonding, hydrophobic interactions, and inclusion complex formation [14,15]. The physical characteristics of starch, such as crystallinity, gelatinization behavior, and enzymatic digestibility, may be greatly altered by these interactions. Meanwhile, the starch may protect phenolic compounds from degradation during thermal processing. Such synergistic effects highlight the potential of starch–phenolic complexes in developing multifunctional delivery systems for food applications [16,17].

Among various starch sources, rice starch has attracted considerable interest due to its unique structural characteristics, including small granule size, bland flavor, white color, hypoallergenicity, digestibility, and suitability for infant and medical foods [18]. Rice starch granules are generally smaller than those of many other cereal starches, providing a larger specific surface area that may facilitate interactions with guest molecules [18]. Additionally, rice starch has been used in infant foods, gluten-free products, biodegradable packaging materials, edible films, and encapsulation systems for bioactive compounds. These applications reflect their technological value as both a structuring agent and a carrier material in food and food-related systems [19,20]. However, effective processing techniques are needed to encourage interactions between functional chemicals and starch.

Extrusion cooking is a widely used food processing technology capable of producing starch-based functional materials. During extrusion processing, raw materials are subjected to intense thermal, mechanical, and shear forces under controlled moisture conditions. These conditions induce starch gelatinization, molecular rearrangement, and structural disruption of crystalline regions. In the meantime, strong mechanical shear promotes close mixing between starch chains and incorporated compounds, potentially enhancing the formation of starch–bioactive complexes [21]. In addition, extrusion-induced structural transformations can significantly influence starch digestibility and the release behavior of encapsulated molecules [22].

Despite growing interest in starch–phenolic complexes, the use of extrusion to incorporate PG into rice starch has received limited attention. In particular, the extent to which PG addition influences the structural reorganization of extruded rice starch, as well as the resulting antioxidant functionality and digestibility, remains insufficiently clarified. A better understanding of these structure–function relationships would help assess whether extrusion can serve as a practical route for preparing starch-based matrices that carry PG. Therefore, this study aimed to prepare rice starch–PG complexes by extrusion cooking and to characterize their structural and functional properties. The effects of PG addition on complex formation were first assessed by the complex index (CI). Multi-scale structural changes in the extruded starch matrix were then analyzed using Small-angle X-ray scattering (SAXS), Fourier-transform infrared spectroscopy (FTIR), carbon-13 nuclear magnetic resonance (^13^C NMR), and X-ray diffraction (XRD), while density functional theory (DFT) calculations were used as a supplementary approach to examine possible molecular interactions between starch fragments and PG. In addition, 1-diphenyl-2-picrylhydrazyl (DPPH) radical-scavenging activity, pasting and rheological properties, and in vitro starch digestibility were evaluated to relate structural changes to potential functionality. The study was designed to clarify how PG incorporation during extrusion modifies the organization and properties of rice starch, rather than to establish a complete molecular mechanism.

## 2. Materials and Methods

### 2.1. Reagents

PG (98%) and rice starch were obtained from Yuanye Biotechnology Co., Ltd. (Shanghai, China) and Beneo-Orafti (Oreye, Belgium), respectively. The GOPOD testing kit was purchased from Megazyme (Bray, Co., Wicklow, Ireland), whereas Sigma-Aldrich (St. Louis, MO, USA) supplied both DPPH and pancreatic α-amylase (A3306). Acetic acid solution, dimethyl sulfoxide (DMSO), ethanol solution (95% and 70%, *v*/*v*), iodine solution, potassium iodide, lithium bromide (LiBr), and sodium hydroxide solution were used in this study.

### 2.2. Extrusion Encapsulation of PG in Starch and Complexing Index

Sample preparation involved blending PG into the rice starch to achieve target concentrations of 0, 25, 50, and 100 mg/g. These mixtures underwent thermomechanical treatment in an extruder (co-rotating, twin-screw, HK-36, Nanjing KY Chemical Machinery Co., Ltd., Nanjing, China; L/D = 52) [23]. The extrusion parameters were set as follows: solid feed rate, 10 kg/h; water feed rate, 2.5 L/h; screw speed, 90 rpm; and barrel temperature profile, 70, 80, 90, 100, and 100 °C from the feeding zone to the die. After emerging from the die, the samples underwent a 24-h drying phase at 45 °C, followed by mechanical grinding and sieving (100-mesh) to obtain standardized powders. Based on the amount of PG added, the control sample devoid of PG was designated ES-PG-0, and the treated samples were designated ES-PG-25, ES-PG-50, and ES-PG-100.

The CI of the samples was quantified following a previously established protocol [24]. Briefly, to achieve full starch gelatinization, 0.2 g of the sample was suspended in 10 mL of deionized water and heated to a boiling temperature for 30 min. Once cooled to ambient temperature, the dispersion was centrifuged at 4000× *g* for 10 min. Next, 4 mL of an aqueous iodine solution (containing 1.3% I_2_ and 2% KI) was reacted with 100 μL of the resulting supernatant. The spectrophotometric absorbance was then recorded at 690 nm, and the CI was computed using Equation (1):

(1) $$\mathrm{CI}\left(\%\right)=\frac{A_{\mathrm{Control}}-A_{\mathrm{Sample}}}{A_{\mathrm{Control}}}\times 100$$

### 2.3. Amylose Content

An iodine–amylose binding principle-based colorimetric assay was used to measure the amylose concentration [25]. First, 9 mL of 1 mol/L sodium hydroxide was added after 100 mg of the material was dissolved in 1.0 mL of 95% ethanol. This suspension was left undisturbed for 10 min prior to being subjected to a boiling water bath for another 10 min. The mixture was diluted with distilled water in a volumetric flask to a final volume of 100 mL when it had cooled to room temperature. For color development, a diluted sample (2.5 mL) was mixed with 0.5 mL of 1 mol/L acetic acid, 1 mL of iodine–potassium iodide reagent (2.5 μmol/L I_2_ and 6.5 μmol/L KI), and 46 mL of distilled water. Following a 20-min reaction period at room temperature, the optical density was measured at 620 nm. A calibration curve, generated simultaneously from repeated dilutions of maize amylopectin and potato amylose standards, was used to determine the final amylose content.

### 2.4. Molecular Weight Distribution

Following previously established protocols [26], size-exclusion chromatography combined with multi-angle laser light scattering and refractive index detection (SEC-MALLS-RI) was used to measure the molecular dimensions of the starches. The SEC-MALLS-RI system consisted of a Waters gel permeation chromatography system equipped with a refractive index detector (Waters Corporation, Milford, MA, USA) and a multi-angle light scattering detector (Waters Corporation) operated at 658 nm. For sample preparation, 5 mg of starch was dispersed in 5 mL of a dimethyl sulfoxide (DMSO) solvent system containing 0.5% (*w*/*w*) LiBr. To ensure complete dissolution, the mixture was heated to 60 °C for 24 h in a thermomixer. The chromatographic analysis was performed using the 0.5% *w*/*w* DMSO/LiBr solution as the mobile phase. Analysis was performed using a 7.8 mm × 300 mm Styragel HMW 7 DMF column and an 8 mm × 300 mm Styragel HMW 6E DMF column (Waters Corporation), with an injection volume of 500 μL. Flow rate 0.3 mL/min, column temperature 50 °C, test wavelength 658 nm, *d*_n_/*d*_c_ = 0.074 mL/g. The weight-average (*M*_w_) and number-average (*M*_n_) molecular weights, alongside the polydispersity index (*M*_w_/*M*_n_), were quantified using the SEC-MALLS-RI system described above.

### 2.5. Small-Angle X-Ray Scattering

SAXS measurements were performed using a SAXS instrument (Anton Paar, Graz, Austria) with a Cu–Kα radiation source operated at 40 kV and 50 mA, in accordance with our previously established protocol [27]. SAXSSquant 2D was used to normalize the raw scattering data, while SAXSSquant 3D was used to do background subtraction. According to Mildner and Hall [28], the Debye–Bueche and power-law equations were used to theoretically predict the ensuing scattering patterns of the complex samples, as follows:

(2) $$I(q)=\frac{I_{\mathrm{DB}}\left(0\right)}{{(1+\varepsilon^{2}q^{2})}^{2}}+{Aq}^{-\delta }$$

$\frac{I_{\mathrm{DB}}\left(0\right)}{{(1+\varepsilon^{2}q^{2})}^{2}}$ denotes the Debye–Bueche intensity function, where the parameter ε reflects the heterogeneous correlation lengths within the starch paste system. For the power-law component ${Aq}^{-\delta }$, A and δ represent the scaling pre-factor and the scattering exponent, respectively.

### 2.6. Fourier-Transform Infrared Spectroscopy

FTIR analysis was conducted using a Bruker Tensor 37 spectrometer (Madison, WI, USA) over the wavenumber range 4000–400 cm^−1^. For each measurement, a total of 64 scans were collected against an air backdrop at a resolution of 4 cm^−1^. To analyze the molecular characteristics, the spectral region from 1200 to 900 cm^−1^ was deconvolved using the Lorentz line-shape function, with an enhancement factor of 1.9 and a half-bandwidth of 19 cm^−1^ [29]. Subsequently, OMNIC software (version 9.2) was used to identify and extract the absorption band intensities at 1045 cm^−1^ and 1022 cm^−1^. These values were used to determine the *R*_1047/1022_ ratio, a reliable indicator of the short-range ordered structure of the samples [30].

### 2.7. Solid ^13^C Cross-Polarization/Magic Angle Spinning NMR

Solid ^13^C CP/MAS NMR spectra were recorded using a Bruker AVANCE III HD 400 spectrometer (Bruker, Madison, WI, USA) equipped with a 4-mm broadband dual-resonance MAS probe. Approximately 500 mg of the sample was placed into the spinner and inserted into the center of the magnetic field. The NMR spectrum with CP and MAS was recorded at 100.613 MHz at 295 K. Over 6000 scans were accumulated for each spectrum, with a recycle delay of 2 s [31,32]. Amorphous starch (AS) and all ES-PG samples were measured under the same conditions. Briefly, the C_4_ resonance at approximately 84 ppm was used as an internal reference. Each sample spectrum was multiplied by a correction factor to align the signal intensity at 84 ppm with that of the AS spectrum. The normalized AS spectrum was then subtracted from the corrected sample spectrum to obtain the difference spectrum corresponding to the helix structures. Peaks at 99–102 ppm in the C_1_ region indicate V-type single helices (eight glucose cycles per turn), and 103.2 ppm is also associated with the AS content at the junction points of amylopectin double helices. In addition, the peaks centered on 101.5, 100.5, and 99.4 ppm in the C_1_ region are characteristic of the double-helical structure of A-type starch [33,34]. The helix structure contents were calculated using the following Equation (3) [31] by using PeakFit software (version 4.12; Systat Software Inc., San Jose, CA, USA):

Helix structures content (%) = A_i_/A_total_ × 100 (3)

where A_i_ is the integrated area of the fitted peak assigned to a specific structural component, and A_total_ is the sum of the integrated areas of all fitted peaks in the C_1_ region. The amorphous content was then calculated by subtracting the sum of the single-helical and double-helical contents from 100%: Amorphous content (%) = 100 − single-helical content (%) − double-helical content (%).

### 2.8. X-Ray Diffraction

Following the protocols established in prior research [27], the samples were prepared and subsequently subjected to XRD measurements using an X’Pert Powder diffractometer (Malvern Panalytical, Almelo, The Netherlands). A 2θ scanning range of 5° to 40° was used to capture the diffraction patterns. To quantify the structural properties, MDI Jade software 6.5 (Materials Data, Livermore, CA, USA) was employed to determine the proportions of A-type [*X*_A_ (%)] and V-type [*X*_V_ (%)] crystalline structures, with the total crystallinity calculated as their sum [*X*_Total_ (%) = *X*_A_ (%) + *X*_V_ (%)].

### 2.9. A Computational Approach to the Interactions Between PG and Starch

The GaussView program was used to build the initial structural models of starch, the PG molecule, and the starch–PG complex. These models were then subjected to geometric optimization and vibrational analysis using the B3LYP/6-31G(d) approach within the DFT framework [35,36]. The difference between the complex’s energy and the total energy of its fragments, known as binding energy (ΔE), represents the interaction energy between starch and PG and is related to the stability of their complexes. It was defined for this system as Equation (4) [37]:

(4) ΔE=E(gua-arc)−[E(gua)+E(arc)]

Here, E (gua-arc) represents the total energy of the starch–PG complex, whereas E (gua) and E (arc) correspond to the energies of the isolated starch and PG molecules, respectively. ΔECP-ZPE denotes the binding energy after correction for both basis set superposition error (BSSE) and zero-point energy (ZPE). In contrast, ΔE refers to the uncorrected binding energy, while ΔZPE and ΔBSSE indicate the energies corrected only for ZPE and BSSE, respectively.

### 2.10. Pasting and Rheological Properties

An MCR302e rotational rheometer, supplied by Anton Paar (Graz, Austria), served as the primary instrument for assessing the pasting characteristics of the 6% *w*/*w* (dry basis) samples’ suspensions [38]. Throughout the measurement, a standard rotation speed of 160 rpm was maintained, preceded only by a higher-speed pre-shear condition at 960 rpm. The 6% suspension was subjected to a programmed temperature sweep: it was initially heated at 5 °C/min from 30 °C to 95 °C, held steady at 95 °C for 30 min, and subsequently cooled back to 50 °C using the same temperature gradient, ending with a final 30 min hold at 50 °C. Consequently, five critical parameters characterizing the pasting process were recorded: setback viscosity (η_sb_), breakdown viscosity (η_bd_), final viscosity (η_fv_), peak viscosity (η_pv_), and pasting temperature (T_p_).

Prior to rheological testing, starch was dispersed in deionized water to yield 0.1 g/mL suspensions, which were subsequently stirred for 20 min (500 rpm, 25 °C). Testing was performed using a 40 mm parallel-plate setup, with silicone oil applied around the measuring gap to prevent sample dehydration. After confirming the linear viscoelastic region through amplitude sweeps, dynamic oscillatory frequency sweeps were conducted at 25 °C using a fixed 1% strain. Over a range of angular frequencies from 1 to 100 rad/s, the storage modulus (G′) and loss modulus (G″) evolution [39].

### 2.11. In Vitro Antioxidant Activity

To evaluate the DPPH radical-scavenging capacity, aqueous solutions of the ES-PG samples and PG were prepared at the following concentrations: 0, 0.1, 0.2, 0.4, 0.6, 0.8, and 1.0 mg/mL. Each dilution was then mixed with 0.4 mL of a 0.4 mM DPPH ethanol solution in an aliquot (0.1 mL). Following a 2-min vortex mixing, the reaction mixtures were incubated in the absence of light on a platform shaker at 200 rpm for 90 min. Optical density was subsequently recorded at 517 nm using a Microplate Reader (Infinity 200Pro, Tecan, Switzerland). The scavenging activity was calculated using Equation (5) [40]:

(5) $$\mathrm{Scavenging}\;\mathrm{activity}\;(\%)=(1-\frac{A_{1}-A_{2}}{A_{0}})\;\times \;100$$

In this equation, A_1_ represents the absorbance of the test mixture (sample with DPPH), A_2_ denotes the background absorbance of the sample alone (where ethanol replaces the DPPH reagent), and A_0_ indicates the absorbance of the blank control (using distilled water in place of the sample).

### 2.12. In Vitro Digestibility Kinetics

The in vitro digestibility assay was performed according to the starch fractionation and kinetic analysis method described by Guo et al. [29] and Butterworth et al. [41], with minor modifications. Sample aliquots (0.5 mL) were collected at 0, 20, 60, 120, and 180 min during the in vitro digestion process. To halt further enzymatic degradation, these aliquots were thoroughly mixed with 20 mL of 70% ethanol. Following a 5-min centrifugation at 4000 rpm, 0.1 mL of the clear supernatant from each sample was combined with 3 mL of GOPOD reagent. This final mixture was kept in a light-protected water bath (45 °C) for a duration of 20 min. Starch nutritional fractions were subsequently determined based on their specific digestion times: resistant starch (RS, >120 min), slowly digestible starch (SDS, 20–120 min), and rapidly digestible starch (RDS, <20 min). Their contents were calculated utilizing the following Equations (6)–(8) [29]:

(6) $$RDS=(G_{20}-FG)\times 0.9$$

(7) $$\mathrm{SDS}=(G_{120}-G_{20})\times 0.9$$

(8) RS=TS−(RDS+SDS)

In these equations, total starch is denoted by TS, whereas the free glucose levels measured at 0, 20, and 120 min are denoted by FG, G_20_, and G_120_, respectively.

The starch-digesting experimental data were fitted to a pseudo-first-order kinetic model, and the fitting quality was evaluated using *R*^2^, expressed as Equation (9):

(9) $$C_{t}=C_{\infty }\left(1-e^{-kt}\right)$$

where the digestion time (min) is defined as *t*, and the reaction’s rate constant is represented by *k* (min^−1^). The variable *C*_t_ indicates the percentage of TS broken down at time *t*, whereas $C_{\infty }$ refers to the ultimate percentage of hydrolysis achieved at the endpoint of the reaction. Characterization of the digestion curves was performed via the logarithm of the slope (LOS) procedure. As outlined by Butterworth et al. [41], the LOS plots were produced using Equation (9), the logarithmic form of the basic model’s first derivative. By plotting this linear regression, the values for *k* and $C_{\infty }$ could be determined directly from the derived slope and intercept using Equation (10):

(10) $$\ln(\frac{dC}{dt})=-\mathrm{kt}+\ln{(C}_{\infty }k)$$

### 2.13. Statistical Analysis

SPSS V23.0 (IBM, Armonk, NY, USA) was used for data processing and statistical analysis. All experiments were performed at least in triplicate. Results are expressed as mean ± standard deviation (SD). Error bars in the figures represent SD. Statistical significance was evaluated by one-way analysis of variance (ANOVA) followed by Fisher’s least significant difference (LSD) test, with *p* < 0.05 considered significant. Principal component analysis (PCA) and Pearson correlation analysis were performed in Origin 2026 (OriginLab, Northampton, MA, USA) to visualize associations among structural parameters, pasting properties, and digestibility-related variables. Because the multivariate analyses were exploratory and performed at the treatment-group level, they were used to indicate associations rather than to establish causality.

## 3. Results and Discussion

### 3.1. Amylose Content and Molecular Weight Distribution

The amylose content and molecular weight parameters of NS and ES-PG-0 are summarized in Table S1. The amylose content of NS was 25.30%, which is consistent with typical values for other native starches [24]. After extrusion, the amylose content significantly increased to 36.90%. The increase stemmed from the disruption of starch chains, particularly the highly branched amylopectin, due to the combined effects of elevated temperature, severe shear forces, and pressure during extrusion [24]. The subsequent production of starch–phenolic complexes is known to be facilitated by such elevated amylose levels.

The molecular weight distribution was also markedly affected by extrusion. As shown in Table S1, NS exhibited an *M*_w_ of 2.75 × 10^7^ g/mol and an *M*_n_ of 1.52 × 10^7^ g/mol. Following extrusion, these values in ES-PG-0 decreased sharply to 0.78 × 10^7^ g/mol and 0.45 × 10^7^ g/mol, respectively. The *M*_w_/*M*_n_, representing the breadth of molecular weight distribution, decreased slightly from 1.81 (NS) to 1.73 (ES-PG-0). These results suggest that extrusion treatment led to significant depolymerization, resulting in a more degraded and slightly more uniform molecular weight distribution [42,43]. These molecular variations are critical because they influence the available chain length for guest-molecule encapsulation.

### 3.2. Complex Index

The CI is a crucial parameter for evaluating the formation efficiency of starch-guest-molecule complexes. As illustrated in Table S1, neither NS nor ES-PG-0 showed complex formation. However, upon the addition of PG, significant complexation was observed. The CI values exhibited a non-linear response to the PG concentration. At a concentration of 25 mg/g (ES-PG-25), the CI was 80.10%. The highest complexation efficiency was achieved in the ES-PG-50 sample, reaching a maximum CI of 88.28%. This suggests that a PG concentration of 50 mg/g provides an optimal ratio for amylose–ligand interaction under the given extrusion conditions. Interestingly, when the PG concentration was further increased to 100 mg/g (ES-PG-100), the CI significantly decreased to 66.06%. The decreased complexation observed at elevated concentrations can be attributed to steric hindrance or phenolic aggregation, which impedes efficient entrapment within the amylose helices during the cooling-induced disorder-to-order structural transition [44,45]. However, because microscopic aggregation, free PG content, and thermodynamic binding parameters were not directly measured in the present study, this explanation should be regarded as a possible mechanism. These results demonstrate that although extrusion is a highly viable approach for fabricating starch–polyphenol complexes, optimizing phenolic acid concentration is paramount to maximizing the final complexation yield.

### 3.3. Fractal Features and Spatial Heterogeneity

**Figure 1:**
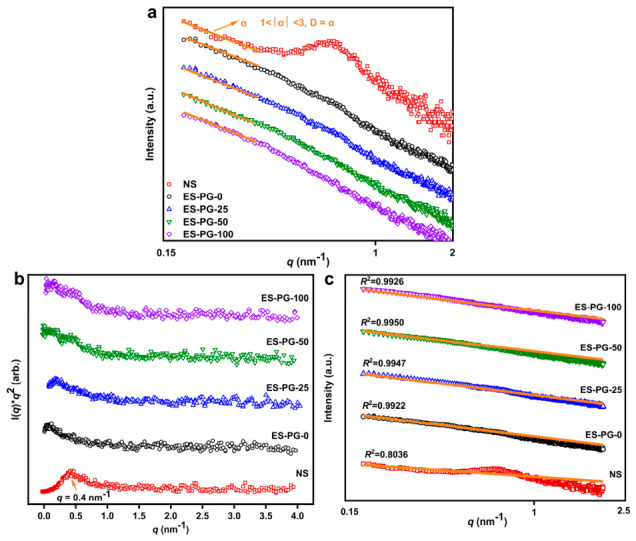
Double-logarithmic SAXS patterns (**a**), Kratky plots (**b**), and SAXS spectra and fit curves (**c**) for NS and ES-PG samples.

SAXS was employed to elucidate structural transitions in starch samples (Figure 1a). NS exhibited a characteristic lamellar peak at *q* ≈ 0.6 nm^−1^, which vanished in all ES-PG samples, confirming the complete destruction of the initial semi-crystalline order [46]. The mass fractal dimension (*D*_m_) derived from the power-law regime (Table 1) decreased from 2.15 (NS) to 1.75 (ES-PG-0), indicating a shift toward a less compact scattering network post-extrusion. However, *D*_m_ progressively increased with PG addition, suggesting that the hydrogen bonding exists in starch–PG interactions promoted molecular densification and the formation of a more compact fractal network.

Kratky plots (*q*^2^*I(*q*) vs. *q*) further revealed the destruction of ordered microdomains, as the prominent peak at *q* = 0.40 nm^−1^ in NS disappeared after extrusion (Figure 1b) [47]. The extruded samples had flattened profiles, indicating considerable macromolecular unfolding, whereas the addition of PG prompted minor structural reorganizations. The interactions between starch and PG, driven by non-covalent forces, re-established locally short-range-ordered domains, thereby reintroducing structural heterogeneity into the amorphous matrix.

To quantify network topology, the Debye–Bueche model was applied (Figure 1c). The model showed high fidelity for extruded pastes (*R*^2^ > 0.99), revealing a continuous decrease in correlation length (ξ) with increasing PG concentration (Table 1) [48]. This reduction in ξ signifies enhanced molecular entanglement and overlap density, suggesting that PG acts as a non-covalent cross-linker that restricts chain mobility and yields a more tightly packed network architecture.

### 3.4. Short-Range Order Structure

**Figure 2:**
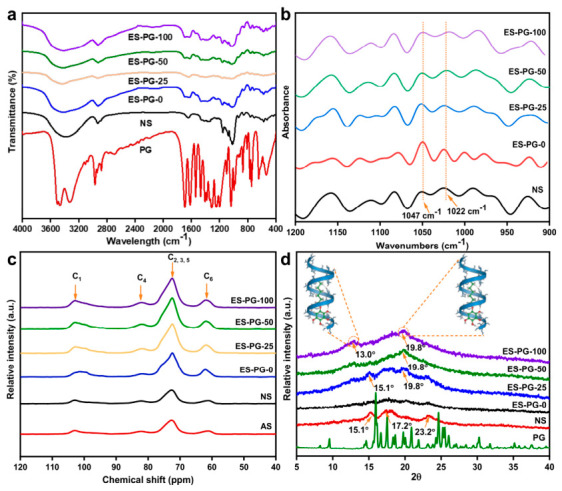
(**a**) ATR-FTIR spectra of PG, NS, and ES-PG samples. (**b**) Deconvoluted ATR-FTIR spectra in the 1200–900 cm^−1^ region of NS and ES-PG samples. (**c**) ^13^C NMR spectra of AS, NS, and ES-PG samples. (**d**) X-ray diffraction patterns of PG, NS, and ES-PG samples.

The FTIR spectra of PG and the ES-PG samples are shown in Figure 2a. Noticeable differences were observed in the regions of 3000–3600, 2800–3000, and 400–1800 cm^−1^. Compared with free PG, the corresponding bands in the ES-PG samples became weaker and smoother, particularly in the fingerprint region. However, no new absorption peaks were clearly observed after extrusion and PG incorporation, suggesting that the association between starch and PG was primarily due to changes in physical interactions rather than the formation of new covalent bonds [24,49].

To examine the alterations in the short-range molecular arrangement of starch after extrusion and complexation with PG, deconvoluted spectra were employed. The spectra shown in Figure 2b correspond to the deconvoluted ATR-FTIR spectra in the 1200–900 cm^−1^ region. The deconvolution parameters are described in Section 2.6, and the intensities of the bands near 1047 and 1022 cm^−1^ were extracted from the deconvoluted spectra to calculate the *R*_1047/1022_ ratio (Table 1). The absorption band near 1047 cm^−1^ is mainly assigned to C–O and C–C stretching vibrations associated with ordered or crystalline starch domains, whereas the band near 1022 cm^−1^ is related to C–O stretching in amorphous starch regions [50]. Thus, the *R*_1047/1022_ ratio was used to evaluate the short-range ordered structure of starch. The NS exhibited an *R*_1047/1022_ value of 0.66, which slightly increased to 0.68 in the ES-PG-0. Interestingly, incorporating PG during extrusion significantly and progressively increased this ratio, reaching a maximum of 0.75 for the ES-PG-50 sample before leveling off at 0.74 for the ES-PG-100 sample. This pronounced enhancement in short-range order suggests that PG facilitates the formation of a highly ordered structural domain at the molecular level. During severe thermomechanical extrusion, native inter- and intramolecular hydrogen bonds are disrupted [51]. However, PG molecules likely engage in novel non-covalent interactions with starch chains, such as hydrophobic and hydrogen-bonding interactions [24]. These interactions may have limited the mobility of the disordered regions and promoted reassembly into more locally ordered domains.

### 3.5. Helical Structure

The structural transformations induced by extrusion and PG complexation were further examined by solid-state ^13^C CP/MAS NMR spectroscopy. As shown in Figure 2c, the main carbon resonances were observed at 96–106 ppm for C_1_, 80–85 ppm for C_4_, 68–78 ppm for C_2_, C_3_, and C_5_, and 58–65 ppm for C_6_ [31]. The amorphous, single-helical, and double-helical structure contents were calculated by the method of Section 2.7 and are listed in Table 1. NS was predominantly composed of double-helical structures (42.49%) and amorphous regions (55.18%), with a negligible single-helix fraction (2.33%). The ES-PG-0 severely disrupted these native crystalline, reducing the double-helix content to 18.01% and increasing the amorphous fraction to 78.18% due to high temperature, moisture, and shear forces [27]. However, the incorporation of PG led to a dramatic, dose-dependent emergence of single-helical structures, surging from 3.81% in ES-PG-0 to an impressive 17.29% in ES-PG-100, while the double-helix content concurrently declined to 10.11%. This remarkable conformational transition provides compelling evidence for the successful formation of starch–polyphenol inclusion complexes. Consistent with previous studies on extrusion, PG acts as an active guest molecule that inserts into the hydrophobic cavity of the amylose helix [52,53]. This complexation thermodynamically stabilizes the single-helical conformation and sterically hinders the re-association of starch chains into double helices during the subsequent cooling phase.

### 3.6. Crystalline Structure

The long-range crystalline characteristics evaluated via XRD perfectly aligned with the short-range and conformational changes (Figure 2d). The XRD pattern of NS exhibited characteristic A-type diffraction peaks at 15.1°, 17.2°, and 23.2°, corresponding to *X*_Total_ of 24.60%. The destructive thermomechanical forces of extrusion caused *X*_Total_ to plummet to 11.26% in ES-PG-0. Concurrently, the emergence of diffraction peaks near 13.0° and 19.8° supports the formation of V-type crystalline structures, suggesting that PG promoted the transformation of part of the disordered amylose chains into single-helical inclusion complexes [40]. The addition of PG led to a partial recovery of total crystallinity, reaching 14.22% in ES-PG-100. Notably, this structural recovery was entirely driven by the formation of V-type crystallinity, which increased substantially from 2.12% in ES-PG-0 to 6.71% in ES-PG-100, while the residual A-type crystallinity continued to decrease to 7.51% (Table 1). The transition from A-type to V-type diffraction patterns conclusively demonstrates that the gelatinization and melting of native crystals during extrusion yield flexible, disorganized amylose chains [54]. These chains then synergistically interact with the hydrophobic aromatic rings of PG, inducing the self-assembly of left-handed single amylose helices that encapsulate the active phenolic molecules [24,53]. This process highlights the robust capacity of extrusion cooking to continuously and efficiently produce stable, long-range ordered V-type crystalline structures with significant potential for targeted delivery and functional food applications.

### 3.7. Interaction Energy

**Figure 3:**
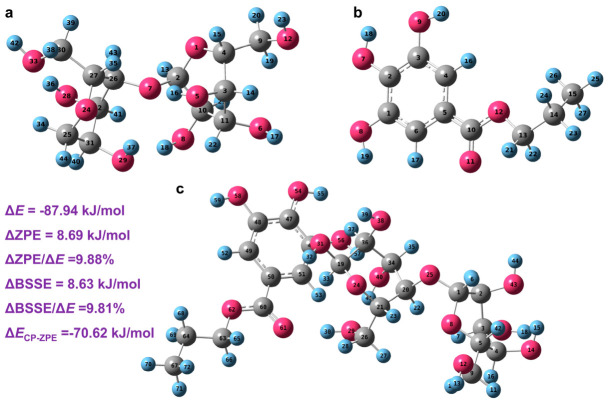
Optimized stable conformer of the glucose dimer-PG system. (**a**–**c**) Glucose dimer (Starch), PG, glucose dimer + PG. The magenta sphere represents the O atom, the gray sphere represents the C atom, and the sky-blue ball represents the H atom.

First, we use DFT calculations to predict the physical molecular interactions between starch and PG via a geometric structural optimization. Figure 3 shows the optimized structures of the glucose dimer (starch) [55], PG, and the starch–PG complex formed from the glucose dimer and PG. Based on previous studies, we calculated the typical configuration of glucose dimer-PG complex [56,57]. As shown in Figure 3c, the BSSE corrections contribute less than 10% of the corresponding uncorrected ΔE values, indicating that the B3LYP/6-31G(d) method achieves a reasonable compromise between computational accuracy and cost for the systems investigated. In contrast, the contribution of ZPE to the uncorrected ΔE ranges from 8.21% to 16.35%, suggesting that the effect of ZPE is more pronounced than that of BSSE in the present calculations, in agreement with previous findings [58]. The negative ΔE values indicate that complex formation between starch and PG is energetically favorable. Notably, the binding energy reaches −70.62 kJ/mol, implying relatively strong intermolecular interactions within the complex. These results suggest that the glucose dimer forms a stable complex with PG via hydrogen-bonding interactions that contribute significantly to the system’s stability [59].

### 3.8. Pasting and Viscoelastic Properties

**Figure 4:**
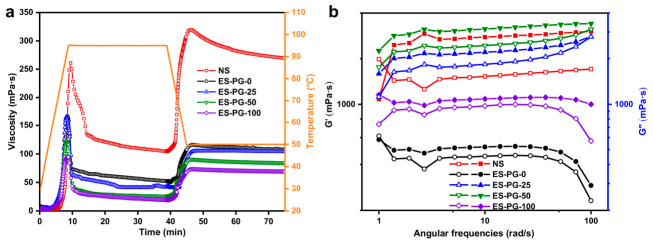
(**a**) Paste curves and (**b**) rheological properties, including the G′ (solid box) and G″ (hollow box), for the NS and ES-PG samples.

The pasting profiles (Figure 4a) reflected the microstructural and thermal transformations of the starch systems. NS exhibited typical pasting behavior, with η_pv_ = 260.81 mPa·s and η_fv_ = 319.22 mPa·s (Table 2). After extrusion treatment, both parameters decreased markedly, with η_pv_ and η_fv_ reduced to 166.74 mPa·s and 115.78 mPa·s, respectively. This decrease is consistent with the well-documented effects of extrusion, in which intense thermomechanical shear disrupts the starch granules’ semi-crystalline structure and promotes the macromolecular depolymerization of amylose and amylopectin chains. As a result, the fragmented starch structure exhibits reduced water-binding capacity, thereby weakening pasting viscosity. The addition of PG during extrusion further altered the thermal and pasting characteristics in a concentration-dependent manner. As the PG level increased from 0 to 100 mg/g, the T_p_ decreased from 69.81 °C to 57.57 °C, while η_pv_ and η_fv_ declined progressively, reaching minimum values of 94.38 mPa·s and 73.31 mPa·s in ES-PG-100. This trend suggests that PG may act as a plasticizing or chain-mobility-modifying agent within the extruded starch matrix [60]. In addition, the η_sb_, which reflects short-term retrogradation, decreased substantially from 214.66 mPa·s in NS to 51.42 mPa·s in ES-PG-100. This decrease may be attributed to the formation of non-covalent contacts between phenolic compounds and leached amylose chains, including hydrogen bonding and hydrophobic interactions [17,51]. These interactions provide steric hindrance, which improves the starch paste’s anti-retrogradation qualities by preventing amylose chains from re-associating into ordered double helices after cooling.

The dynamic oscillatory rheological results (Figure 4b) revealed the viscoelastic behaviors and microstructural changes in the starch systems. The creation of a mostly elastic, solid-like gel network was indicated by the G′ continuously being greater than the G″ for all samples over the ω range. However, the incorporation of PG produced a biphasic response in the dynamic moduli. Both G′ and G″ initially increased with PG addition and reached maximum values at 50 mg/g, where the ES-PG-50 sample exhibited the highest viscoelastic moduli, even exceeding those of the unextruded NS. This enhancement suggests that, at moderate concentrations, PG acts as a physical cross-linking agent. The multiple hydroxyl groups of PG can form extensive intermolecular hydrogen bonds with depolymerized starch chains, effectively bridging fragmented molecules and reinforcing the three-dimensional gel network [61]. Nevertheless, further increasing the PG concentration to 100 mg/g resulted in a decline in both G′ and G″. This reduction indicates that excessive PG disrupts starch–starch entanglements. When present above a critical threshold, phenolic molecules may act as diluents rather than cross-linkers, generating steric repulsion or localized phase separation, ultimately weakening the structural integrity and continuity of the viscoelastic gel network [62].

Compared with previously reported extruded starch–phenolic acid systems, in which phenolic incorporation generally reduced paste viscosity while increasing ordered or resistant fractions [24,51], the present rice starch–PG system showed a similar viscosity reduction but a clearer optimum in viscoelastic reinforcement at 50 mg/g PG. This biphasic response is beneficial because it indicates that PG can simultaneously contribute antioxidant functionality and strengthen the weak gel network at an appropriate loading level, whereas excessive PG weakens the network. In contrast to cast or non-extruded starch–polyphenol complexes, extrusion provides intensive mixing, gelatinization, and molecular disruption in a continuous process, which may increase contact between amylose chains and PG, thereby favoring the formation of single-helical and V-type ordered domains [17,53].

### 3.9. Antioxidant Activity

**Figure 5:**
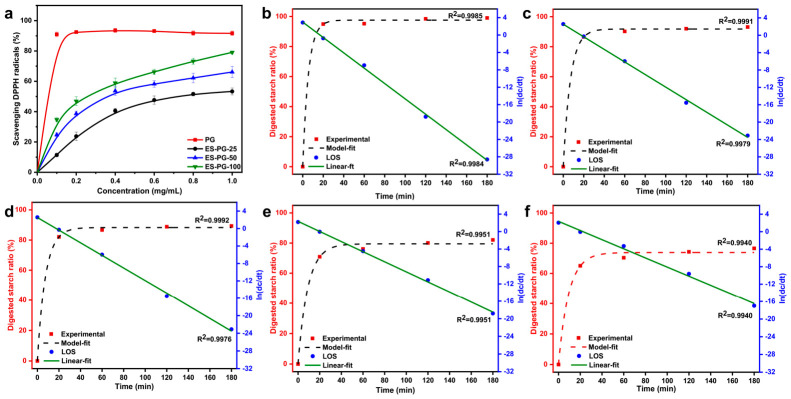
(**a**) DPPH radicals scavenging activity of PG and ES-PG samples. (**b**–**f**) In vitro starch digestion curves fitted using the pseudo-first-order exponential model and the corresponding LOS plots for NS, ES-PG-0, ES-PG-25, ES-PG-50, and ES-PG-100.

The antioxidant functionalization of the ES-PG samples was evaluated by measuring their ability to scavenge DPPH radicals. As depicted in Figure 5a, the pure PG sample exhibited rapid and highly efficient free radical scavenging. Upon starch extrusion, the resulting ES-PG matrices demonstrated a clear dose-dependent improvement in antioxidant capacity. The scavenging activity of the extruded samples increased proportionally with the PG incorporation level, with ES-PG-100 > ES-PG-50 > ES-PG-25. These results indicate that the bioactive properties of PG were successfully retained during the high-temperature, high-shear extrusion process, thereby conferring measurable radical-scavenging functionality on the starches [63]. It should be noted that the present DPPH assay reflects antioxidant activity after a fixed reaction period rather than long-term antioxidant-release kinetics. Therefore, these data should not be interpreted as evidence of sustained antioxidant protection during storage. Compared with conventional starch–polyphenol systems prepared by non-extrusion complexation, the present extruded system’s main advantage is its ability to retain measurable PG activity while simultaneously modifying starch structure and digestibility. Future work should include time-resolved antioxidant measurements over 24, 48, and 96 h, and preferably longer periods relevant to target food applications, to distinguish rapidly released/free PG from slowly released or complex-protected PG.

### 3.10. Analysis for In Vitro Digestibility and First-Order Kinetics

The nutritional profiles of the ES-PG samples were characterized by quantifying the RDS, SDS, and RS contents (Table 2). NS displayed a highly digestible profile, characterized by an overwhelmingly high RDS content (94.89%) and negligible RS (1.59%). Interestingly, the ES-PG-0 significantly altered the digestive profile, reducing the RDS to 86.52% and increasing the RS to 8.09% (*p* < 0.05). More importantly, the co-extrusion of starch with PG induced a profound, concentration-dependent restriction on enzymatic hydrolysis. As the PG concentration increased from 25 to 100 mg/g, the RDS fraction declined significantly and continuously, reaching a minimum of 65.29% in the ES-PG-100 sample. Concurrently, the RS content increased dramatically, peaking at 25.78% in ES-PG-100, substantially higher than both the native and control extruded starches. The SDS fraction also broadened incrementally from 3.52% in NS to 8.93% in ES-PG-100.

These results compare favorably with other extrusion-assisted starch–phenolic systems, where reduced starch digestibility is commonly attributed to phenolic-induced ordering, hydrogen bonding, and the formation of enzyme-resistant single-helical structures [24,51]. The benefit of the rice starch–PG system is that the reduction in digestibility occurred together with retained antioxidant activity and V-type structural development, suggesting a multifunctional matrix rather than a solely digestibility-modified starch. Austin et al. [64] also noted that tannins were more effective at reducing starch digestibility when cooked with starch, supporting the importance of thermal processing for starch–phenolic interactions. Thus, PG incorporation during extrusion may enhance resistant starch characteristics by reducing enzyme accessibility via ordered domains and noncovalent starch–PG associations.

To further elucidate the macroscopic digestion behavior, LOS plots were applied to the in vitro digestion curves (Figure 5b–f). All examined samples, from NS (Figure 5b) to ES-PG-100 (Figure 5c–f), showed high linear correlations (*R*^2^ > 0.9940) in the LOS plots, suggesting that enzymatic digestion followed pseudo-first-order kinetics. The derived kinetic parameters, specifically the *k* and the equivalent value at the digestion endpoint (*C*_∞_), quantitatively support the inhibitory effect of PG. The *k* and *C*_∞_ reported in Table 2 were calculated from LOS linear regression. Nonlinear fitting of the original digestion curves to the pseudo-first-order equation was also performed to visualize the fitting performance and to verify whether the digestion data followed first-order behavior. Differences in *R*^2^ values between nonlinear fitting and LOS linearization should be interpreted as differences in model-fitting performance rather than direct evidence of different digestion-rate constants. NS exhibited the most rapid digestion rate (*k* = 0.18 min^−1^) and the greatest overall extent of digestion (*C*_∞_ = 97.54%). However, the inclusion of PG incrementally suppressed the digestion kinetics. In the ES-PG-100 sample, the rate constant *k* was significantly decelerated to 0.11 min^−1^, and the maximum digestion extent *C*_∞_ was restricted to 73.86% (*p* < 0.05). The simultaneous reduction in *k*, *C*_∞_, and RDS, coupled with the increase in RS, strongly suggests that incorporating PG during extrusion structurally fortifies the starch, likely impeding the accessibility and binding affinity of digestive enzymes [51]. Recent studies indicate that increased polyphenol incorporation reduces starch digestibility, as the hydroxyl groups of polyphenols interact with starch via noncovalent bonds, thereby enhancing crystallinity [40]. Additionally, polyphenols trapped within the branched structures of amylose and amylopectin form complexes that resist enzymatic hydrolysis, resembling the enzyme resistance of type 5 RS (RS_5_) [65]. This suggests that polyphenol integration may enhance starch functionality by promoting RS formation.

### 3.11. Pearson Correlation Analysis and Principal Component Analysis

**Figure 6:**
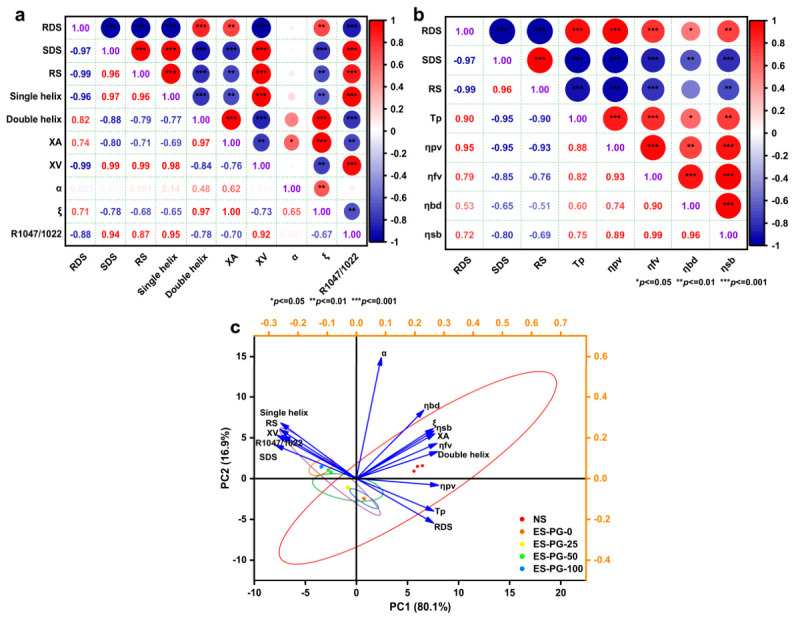
Correlation analysis of starch digestibility with pasting properties (**a**) and multi-scale structures (**b**) of NS and ES-PG samples. (**c**) PCA of pasting properties, multi-scale structural characteristics, and digestibility of NS and ES-PG samples.

Pearson correlation analysis was used as an exploratory tool to relate the structural parameters measured in the present starch–PG samples to the digestibility fractions (Figure 6a). No external polymer system was used for the correlation analysis; all variables were obtained from NS and the ES-PG samples. The proportion of single-helical conformations exhibited a strong positive correlation with both SDS (*r* = 0.97) and RS (*r* = 0.96), while demonstrating a strong negative correlation with RDS (*r* = −0.96). Similarly, indicators of short-range molecular order and relative crystallinity, namely the *R*_1047/1022_ ratio and XV, demonstrated strong positive correlations with SDS and RS (*r* ≥ 0.87). These correlations indicate that the formation of single helices and the concomitant enhancement of short-range ordered structures were associated with slower enzymatic hydrolysis. Mechanistically, the densification of starch through these ordered crystalline and helical domains may induce steric hindrance, restricting the diffusion and active-site binding of digestive enzymes. Because the number of treatment groups was limited, the correlation analysis should be interpreted as supportive evidence of structure–function associations rather than proof of causality.

The hydration and swelling behaviors of starch during thermal processing are macroscopic manifestations of its internal molecular interactions, which concomitantly govern digestibility (Figure 6b). Correlation analysis revealed that RDS showed a strong negative relationship with both SDS (*r* = −0.97) and RS (*r* = −0.99), while exhibiting a high positive correlation with T_p_ (*r* = 0.90). Conversely, SDS and RS were inversely correlated with T_p_ (*r* = −0.95 and −0.90, respectively). The inverse relationship between pasting properties and resistant fractions (SDS/RS) suggests that the same intramolecular and intermolecular forces, such as strong hydrogen bonds within ordered regions, that confer resistance to enzymatic degradation also limit granule swelling and water uptake during heating. Although starch matrices that require more energy to paste (elevated T_p_) typically exhibit a highly restricted amorphous background, the positive correlation between higher T_p_ and higher RDS in these ES-PG modifications suggests that the modification process may disrupt the native granular integrity, thereby facilitating rapid enzymatic degradation once gelatinization is initiated.

To holistically interpret the complex interplay among multi-scale structure, macroscopic functionality, and nutritional profiles, PCA was used (Figure 6c) [66]. The first two principal components captured the overwhelming majority of the system’s variance, with PC1 and PC2 accounting for 80.1% and 16.9%, respectively. The loading figure showed a clear mechanical grouping in which parameters related to enzymatic resistance and ordered structure, including single helix, *X*_V_, SDS, RS, and *R*_1047/1022_, were closely clustered on the negative axis of PC1. Conversely, RDS and pasting properties (such as T_p_, η_pv_, and η_fv_) loaded heavily on the positive axis of PC1. Concurrently, the scores plot demonstrated a distinct spatial migration of the sample groups (NS, ES-PG-0, ES-PG-25, ES-PG-50, and ES-PG-100) across the principal components. Together with the Pearson analysis, PCA supports the view that PG-containing extruded samples shifted from a rapidly digestible and highly pasting starch matrix toward a more ordered, sterically restricted matrix with increased SDS/RS fractions. This multivariate analysis therefore complements, rather than replaces, the direct structural measurements obtained from CI, FTIR, ^13^C NMR, and XRD.

## 4. Conclusions

In summary, this study showed that twin-screw extrusion can promote the association of rice starch with PG and generate starch–PG complexes with distinct structural and functional characteristics. Extrusion disrupted the native semi-crystalline organization of rice starch and reduced molecular size, creating conditions that favored subsequent PG incorporation. Among the tested formulations, the sample containing 50 mg/g PG showed the highest CI, indicating that the level of guest addition strongly affected complex formation. Structural analyses consistently showed that PG incorporation was associated with increased short-range order, greater single-helical content, and the emergence of V-type crystalline domains in the extruded starch. The computational results further supported the possibility that hydrogen bonding contributed to the stabilization of the starch–PG association. These structural changes were accompanied by lower pasting viscosity, altered viscoelastic behavior, retained radical-scavenging activity, and slower in vitro starch hydrolysis, with the resistant starch content increasing to 25.78%. Overall, the results suggest that extrusion is a feasible approach for preparing rice starch-based matrices containing PG and for modulating both antioxidant functionality and digestibility-related properties. In addition, starch recovered from rice-processing by-products or starch-rich food waste streams may provide a sustainable, low-cost starting material for preparing modified starch-based delivery systems. However, the present study is limited by its reliance on in vitro digestion, a fixed-time DPPH antioxidant assay, and model-based structural interpretation. Future work should examine PG release under gastrointestinal conditions, time-resolved antioxidant activity over 24, 48, and 96 h, and longer storage-relevant periods, in vivo bioavailability, stability during processing and storage, and functional performance in real food matrices. Comparative studies with other phenolics, such as gallic acid, catechin, and quercetin, will be needed to determine whether the observed complexation behavior extends to broader starch–polyphenol systems.

## Figures and Tables

**Table 1:** The fractal dimension (α = *D*_m_), parameters (ξ) from Debye–Bueche and Power-law, short-range ordered degree (*R*_1047/1022_), helical, and crystallinity structures for NS and ES-PG samples.

Samples	α = *D*_m_	ξ (nm)	*R*_1047/1022_	Amorphous	Single Helix	Double Helix	*X*_Total_ (%)	*X*_A_ (%)	*X*_V_ (%)
NS	2.15 ± 0.01 *^a^*	6.47 ± 0.02 *^a^*	0.66 ± 0.01 *^b^*	55.18 ± 5.33 *^e^*	2.33 ± 0.17 *^e^*	42.49 ± 5.16 *^a^*	24.60 ± 0.25 *^a^*	24.09 ± 0.14 *^a^*	0.51 ± 0.11 *^e^*
ES-PG-0	1.75 ± 0.02 *^d^*	1.97 ± 0.01 *^b^*	0.68 ± 0.02 *^b^*	78.18 ± 0.33 *^a^*	3.81 ± 0.19 *^d^*	18.01 ± 0.14 *^b^*	11.26 ± 0.22 *^c^*	9.14 ± 0.11 *^b^*	2.12 ± 0.11 *^d^*
ES-PG-25	1.83 ± 0.02 *^c^*	1.87 ± 0.04 *^c^*	0.73 ± 0.01 *^a^*	73.43 ± 0.26 *^b^*	10.12 ± 0.11 *^c^*	16.45 ± 0.15 *^c^*	11.59 ± 0.24 *^c^*	8.18 ± 0.12 *^c^*	3.41 ± 0.12 *^c^*
ES-PG-50	2.00 ± 0.03 *^b^*	1.78 ± 0.02 *^d^*	0.75 ± 0.02 *^a^*	72.74 ± 0.25 *^c^*	16.10 ± 0.12 *^b^*	11.16 ± 0.13 *^d^*	13.91 ± 0.22 *^b^*	8.10 ± 0.10 *^c^*	5.81 ± 0.12 *^b^*
ES-PG-100	2.04 ± 0.02 *^b^*	1.77 ± 0.04 *^d^*	0.74 ± 0.04 *^a^*	72.60 ± 0.26 *^d^*	17.29 ± 0.11 *^a^*	10.11 ± 0.15 *^e^*	14.22 ± 0.23 *^b^*	7.51 ± 0.11 *^d^*	6.71 ± 0.12 *^a^*

NS: native starch; ES-PG-0, ES-PG-25, ES-PG-50, and ES-PG-100: Extruded starch with PG at concentrations of 0, 25, 50, and 100 mg/g, respectively. Significant differences exist between values with different letters in the same columns (*p* < 0.05).

**Table 2:** Pasting parameters, RDS, SDS, RS contents, *C*_∞_, and *k* for NS and ES-PG samples.

Samples	T_p_ (°C)	η_pv_ (mPa·s)	η_fv_ (mPa·s)	η_bd_ (mPa·s)	η_sb_ (mPa·s)	RDS (%)	SDS (%)	RS (%)	*k* (min^−1^)	*C*_∞_ (%)
NS	72.41 ± 1.31 *^a^*	260.81 ± 9.80 *^a^*	319.22 ± 12.11 *^a^*	127.13 ± 7.21 *^a^*	214.66 ± 11.51 *^a^*	94.89 ± 1.23 *^a^*	3.52 ± 0.32 *^e^*	1.59 ± 0.91 *^e^*	0.18 ± 0.01 *^a^*	97.54 ± 3.96 *^a^*
ES-PG-0	69.81 ± 0.99 *^b^*	166.74 ± 0.51 *^b^*	115.78 ± 3.56 *^b^*	94.09 ± 0.89 *^b^*	64.91 ± 1.50 *^b^*	86.52 ± 0.62 *^b^*	5.39 ± 0.21 *^d^*	8.09 ± 0.41 *^d^*	0.15 ± 0.02 *^b^*	91.78 ± 2.69 *^b^*
ES-PG-25	62.12 ± 1.51 *^c^*	164.01 ± 0.32 *^c^*	104.91 ± 2.10 *^c^*	93.52 ± 0.21 *^b^*	62.44 ± 0.83 *^c^*	82.03 ± 0.33 *^c^*	6.68 ± 0.11 *^c^*	11.29 ± 0.12 *^c^*	0.14 ± 0.01 *^b^*	88.28 ± 1.65 *^c^*
ES-PG-50	58.34 ± 0.32 *^d^*	121.33 ± 2.50 *^d^*	90.55 ± 1.09 *^d^*	97.51 ± 0.80 *^c^*	59.35 ± 1.42 *^d^*	71.72 ± 1.32 *^d^*	8.36 ± 0.22 *^b^*	19.92 ± 1.10 *^b^*	0.12 ± 0.01 *^bc^*	79.54 ± 2.43 *^d^*
ES-PG-100	57.57 ± 0.42 *^d^*	94.38 ± 3.90 *^e^*	73.31 ± 5.12 *^e^*	99.98 ± 0.41 *^d^*	51.42 ± 1.09 *^e^*	65.29 ± 1.83 *^e^*	8.93 ± 0.12 *^a^*	25.78 ± 1.71 *^a^*	0.11 ± 0.01 *^c^*	73.86 ± 1.47 *^e^*

Setback viscosity (η_sb_), breakdown viscosity (η_bd_), final viscosity (η_fv_), peak viscosity (η_pv_), and pasting temperature (T_p_); *C*_∞_, the equivalent value at the endpoint; *k* (min^−1^), the digestion rate constant; SDS, slowly digestion starch; RS, resistant starch; RDS, rapidly digestion starch; NS: native starch; ES-PG-0, ES-PG-25, ES-PG-50, and ES-PG-100: Extruded starch with PG at concentrations of 0, 25, 50, and 100 mg/g, respectively. Significant differences exist between values with different letters in the same columns (*p* < 0.05).

## Data Availability

The original contributions presented in this study are included in the article/Supplementary Material. Further inquiries can be directed to the corresponding author.

## Supplementary Materials

The following supporting information can be downloaded at: https://www.mdpi.com/article/10.3390/foods15111880/s1, Table S1: The molecular weight distribution and amylose content of ES-PG-0 and NS.

## Abbreviations

The following abbreviations are used in this manuscript:

ANOVA	One-way analysis of variance
BSSE	Basis set superposition error
CI	Complex index
^13^C NMR	Carbon-13 nuclear magnetic resonance
DFT	Density functional theory
DMSO	Dimethyl sulfoxide
DPPH	1-diphenyl-2-picrylhydrazyl
FTIR	Fourier-transform infrared spectroscopy
LOS	Logarithm of the slope
LSD	Least significant difference
*M*_n_	Number-average
*M*_w_	Weight-average
NS	Native starch
PCA	Principal component analysis
PG	Propyl gallate
RDS	Rapidly digestible starch
RS	Resistant starch
SAXS	Small-angle X-ray scattering
SDS	Slowly digestible starch
XRD	X-ray diffraction
ZPE	Zero-point energy
