# Supplementary material for: Extrusion-Assisted Formation of Rice Starch–Propyl Gallate Complexes: Structural Characteristics, Antioxidant Activity, and In Vitro Digestibility
Source: Foods. 2026 May 26;15(11):1880. doi: 10.3390/foods15111880 (PMC13257332; doi:10.3390/foods15111880)
Supplement: Supplementary file 1 [file foods-15-01880-s001.zip › foods-4270215-supplementary.pdf]

# – Supplementary material –

Extrusion-assisted formation of rice starch-propyl gallate complexes: Structural characteristics, antioxidant activity, and *in vitro* digestibility

Simeng Ma [1, †], Zhuanghong Wang [1, †], Honghao Fan [2], Hai He [1, *]

[1] Key Laboratory of Tropical Translational Medicine of the Ministry of Education, School of Public Health, Hainan Academy of Medical Sciences, Hainan Medical University, Haikou 571199, Hainan Province, China.

[2] NJUST-YX Artificial Intelligence Biomedical Technology Innovation Center, Nanjing University of Science and Technology, Nanjing 210094, Jiangsu Province, China.

[†] Simeng Ma and Zhuanghong Wang contributed equally to this work.

[*] **Corresponding authors:** Hai He.

Email address: h.hai@muhn.edu.cn.

Table S1. The molecular weight distribution and amylose content of ES-PG-0 and NS.

Sample	Amylose (%)	M_w (×10^7 g/mol)	M_n (×10^7 g/mol)	M_w/M_n	CI (%)
NS	25.30±1.06^b	2.75	1.52	1.81	-
ES-PG-0	36.90±2.24^a	0.78	0.45	1.73	-
ES-PG-25	-	-	-	-	80.10±1.05^b
ES-PG-50	-	-	-	-	88.28±1.26^a
ES-PG-100	-	-	-	-	66.06±1.13^c

M_n: Number-average Molecular Weight, M_w: Weight-average Molecular Weight; CI: Complex index; NS: native starch; ES-PG-0, ES-PG-25, ES-PG-50, and ES-PG-100: Extruded starch with PG at concentrations of 0, 25, 50, and 100 mg/g, respectively.

Significant differences exist between values with different letters in the same columns ($p < 0.05$).
